# Whole genome sequence-based molecular characterization of blood isolates of carbapenem-resistant *Enterobacter cloacae* complex from ICU patients in Kolkata, India, during 2017–2022: emergence of phylogenetically heterogeneous *Enterobacter hormaechei* subsp. *xiangfangensis*

**DOI:** 10.1128/spectrum.03529-23

**Published:** 2024-02-22

**Authors:** Gourab Halder, Bhaskar Narayan Chaudhury, Subhranshu Mandal, Priyanka Denny, Deotima Sarkar, Mandira Chakraborty, Ujjwayini Ray Khan, Soma Sarkar, Bedobroto Biswas, Arindam Chakraborty, Sourav Maiti, Shanta Dutta

**Affiliations:** 1 Division of Bacteriology, ICMR-National Institute of Cholera and Enteric Diseases, Beliaghata, Kolkata, India; 2 Division of Microbiology, Peerless Hospitex Hospital, Panchasayar, Kolkata, India; 3 Division of Microbiology, CNCI, Rajarhat, New Town, Kolkata, India; 4 Collaborative Research Center for Infectious Diseases in India, Okayama University, JICA Building, ICMR-National Institute of Cholera and Enteric Diseases, Kolkata, India; 5 Division of Microbiology, Calcutta Medical College, College Square, Kolkata, India; 6 Division of Microbiology, Apollo Gleneagles Hospital, Phool Bagan, Kolkata, India; 7 Division of Microbiology, NRS Medical College, Sealdah, Kolkata, India; 8 Division of Microbiology, Desun Hospital, Kasba, Kolkata, India; 9 Division of Microbiology, Fortis Hospital, Anandapur, Kolkata, India; 10 Division of Microbiology, Ruby General Hospital, Kasba, Kolkata, India; Central Texas Veterans Health Care System, Temple, Texas, USA

**Keywords:** *Enterobacter xiangfangensis*, carbapenem resistance, integrons, efflux pumps, WGS, wg-MLST, PFGE, India

## Abstract

Blood-borne infections caused by the carbapenem-resistant *Enterobacter cloacae* complex (CR-ECC) are major public threats with respect to the challenges encountered during treatment. This study describes the whole genome sequencing-based molecular characteristics of blood isolates (*n* = 70) of CR-ECC from patients admitted to the intensive care unit of tertiary care hospitals in Kolkata, India, during 2017–2022 with respect to species identification, antimicrobial resistance (AMR) profiling, mechanism of drug resistance, and molecular subtypes. Vitek2 MALDI and species-specific PCR identified *Enterobacter hormaechei* subsp. *xiangfangensis* (47.14%) as the emerging CR-ECC subspecies in Kolkata. The predominating carbapenemase and extended-spectrum β-lactamase genes found were *bla*_NDM-1_ (51.42%) and *bla*_CTX-M-15_ (27%), respectively. Besides, *bla*_NDM-4_, *bla*_NDM-5_*, bla*_NDM-7_*, bla*_CMH-3_, *bla*_SFO-1_, *bla*_OXA-181_, *bla*_OXA-232_, *bla*_KPC-3_, and *bla*_DHA-7_ genes were also detected, which were not previously reported from India. A multitude of Class 1 integrons (including In180, In4874, In4887, and In4888, which were novel) and plasmid replicon types (IncFIB, IncFII, IncX3, IncHI1-HI2, IncC, and IncR) involved in AMR dissemination were identified. Reverse transcription-PCR and western blot revealed that carbapenem resistance in non-carbapenemase-producing CR-ECC isolates was contributed by elevated levels of *ampC*, overexpression of *acrAB,* and loss of *ompF*. A total of 30 distinct sequence types (STs) were ascertained by multi-locus sequence typing; of which, ST2011, ST2018, ST2055, ST2721, and ST2722 were novel STs. Pulsed-field gel electrophoresis analysis showed heterogeneity (69 pulsotypes with a similarity coefficient of 48.40%) among the circulating isolates, suggesting multiple reservoirs of infections in humans. Phylogenetically and genetically diverse CR-ECC with multiple AMR mechanisms mandates close monitoring of nosocomial infections caused by these isolates to forestall the transmission and dissemination of AMR.

**IMPORTANCE:**

The emergence and extensive dissemination of the carbapenem-resistant *Enterobacter cloacae* complex (CR-ECC) have positioned it as a critical nosocomial global pathogen. The dearth of a comprehensive molecular study pertaining to CR-ECC necessitated this study, which is the first of its kind from India. Characterization of blood isolates of CR-ECC over the last 6 years revealed *Enterobacter hormaechei* subsp. *xiangfangensis* as the most prevalent subsp., exhibiting resistance to almost all antibiotics currently in use and harboring diverse transmissible carbapenemase genes. Besides the predominating *bla*_NDM-1_ and *bla*_CTX-M-15_, we document diverse carbapenemase and *AmpC* genes, such as *bla*_NDM-4_, *bla*_NDM-7_, *bla*_OXA-181_, *bla*_OXA-232_, *bla*_KPC-3_, *bla*_CMH-3_, *bla*_SFO-1_, and *bla*_DHA-7_, in CR-ECC, which were not previously reported from India. Furthermore, novel integrons and sequence types were identified. Our findings emphasize the need for strengthened vigilance for molecular epidemiological surveillance of CR-ECC due to the presence of epidemic clones with a phylogenetically diverse and wide array of antimicrobial resistance genes in vulnerable populations.

## INTRODUCTION

*Enterobacter* spp., the indigenous gut commensals, are Gram-negative, non-spore-forming, rod-shaped, and facultative anaerobe of the family *Enterobacteriaceae* (1). The “ESKAPE” consortium (*Enterococcus faecium*, *Staphylococcus aureus*, *Klebsiella pneumoniae*, *Acinetobacter baumannii*, *Pseudomonas aeruginosa*, and *Enterobacter* spp.) has emerged as major nosocomial pathogens in recent decades (2). Of the 19 species of Enterobacter genus (3), *Enterobacter cloacae* complex (ECC) comprising seven species, namely *E. cloacae*, *Enterobacter asburiae*, *Enterobacter hormaechei*, *Enterobacter kobei*, *Enterobacter ludwigii*, *Enterobacter mori*, and *Enterobacter nimipressuralis*, causes 65%–75% of all hospital-acquired infections (4, 5). After *Escherichia coli* and *K. pneumoniae*, ECC is the third most common contributor to nosocomial infections like pneumonia, urinary tract infections, meningitis, and septicemia globally (6). It is the third most prevalent *Enterobacteriaceae* family member causing bloodstream infection (BSI) in India (7).

Multidrug resistance is a common trait in ECC globally, partly due to “intrinsic resistance” to β-lactam drugs and the ability to rapidly acquire mobile genetic elements through horizontal transfer (6). The ECC members are the second most common β-lactam-resistant genus in *Enterobacteriaceae* (8). ECC are resistant to penicillins and first- and second-generation cephalosporins due to efflux pumps and an inducible chromosomal AmpC-type (class C) cephalosporinase (9). Resistance to third-generation cephalosporins and aztreonam is often caused by mutations in ampD, resulting in constitutive hyperproduction (derepression) of AmpC and plasmid-mediated extended-spectrum β-lactamases (ESBLs) such as *bla*_TEM_, *bla*_SHV_, and *bla*_CTX-M_ (9). Due to rising resistance to aminoglycosides, fluoroquinolones, and third-generation cephalosporins, the carbapenem group (meropenem, imipenem, and ertapenem) “last-resort antibiotics,” have replaced them in multi drug resistant (MDR) ECC treatment (9). However, carbapenem resistance in ECC has risen expeditiously in the last decade, drawing clinical attention. ECC acquire carbapenem resistance by acquiring plasmid-encoded carbapenemase genes (such as *bla*_OXA-48_-like variants, *bla*_VIM_, *bla*_IMP_, *bla*_KPC_, and *bla*_NDM-1_) and the constitutively overexpressing AmpC (especially the *bla*_ACT-type_) and/or ESBL in conjunction with a disruption in membrane permeability [the loss or reduction of the outer membrane proteins (OMPs) OmpF and/or OmpC] and efflux pumps (5, 10, 11). Recently, a novel plasmid-mediated *bla*_CMH-3_ gene has also been discovered, which is closely related to chromosomal *bla*_ACT_ AmpC-type β-lactamases (12).

In clinical settings, ESBL genes like *bla*_TEM_, *bla*_SHV_, and *bla*_CTX-M_ types are frequently observed (13). Over the last decade, reports of a rare ESBL gene *bla*_SFO-1_ (a plasmid-mediated non-TEM, non-SHV, and non-CTX-M class A ESBL) existing alongside carbapenemase genes have gained importance. *bla*_SFO-1_ is an inducible β-lactamase, capable of hydrolyzing cefotaxime very efficiently and ceftazidime poorly and is apparently inactive toward cephamycins and carbapenems (14).

The pathogenicity of *Enterobacteriaceae* can be enhanced by introducing additional virulence factors in addition to carbapenem resistance (15). Like other Enterobacterales, ECC strains have multiple secretion systems (OMPs), siderophores, biofilm mediators, heavy metal resistance, and stress response mechanisms (6, 15). Unlike other Enterobacterales, however, only a few of ECC members have undergone a complete genome-wide analysis, characterizing specific virulence-associated genetic determinants.

Currently, whole genome sequencing (WGS) has become an indispensable tool for generating in-depth epidemiological data on genomic diversity, bacterial distributions, accurate species identification (3), antimicrobial resistance (AMR) gene profiles, and molecular subtypes. Diversity in AMR genes, sequence types (STs), and wide taxonomic varieties among ECC were observed through worldwide surveillance. Additionally, carbapenem-resistant *Enterobacter cloacae* complex (CR-ECC) exhibited regional distribution, with *bla*_NDM_ predominating in China, *bla*_VIM_ and *bla*_OXA-48_ in Europe, and *bla*_KPC_ in North America (9). However, there is a dearth of data from the Indian subcontinent. This study, therefore, focuses on the molecular mechanisms of AMR, plasmid profiles, and molecular subtypes of ECC isolated from hospitalized patients in Kolkata with special emphasis on resistance to carbapenems. This study will help researchers comprehend the burden of AMR caused by this newly discovered carbapenem-resistant pathogen, which is crucial for developing effective treatment and stricter infection prevention and control measures.

## RESULTS

### CR-ECC subspecies

A total of 70 CR-ECC blood isolates obtained from eight hospitals in Kolkata during January 2017 and December 2022 were included in this study. The rate of isolation of CR-ECC from blood samples in Kolkata cannot be ascertained because the total number of samples screened in each hospital was inaccessible. The 16S rRNA PCR and species-specific multiplex PCR identified *E. hormaechei* (*n* = 42) as the predominating CR-ECC among blood isolates, followed by *E. cloacae* (*n* = 22). The most common subspecies of *E. hormaechei* was found to be *E. hormaechei* subsp. *xiangfangensis (Enterobacter xiangfangensis)* (*n* = 33). In addition, *E. hormaechei* subsp. *steigerwaltii (Enterobacter steigerwaltii)* (*n* = 8) and *E. hormaechei* subsp. *hoffmannii* (*n* = 1) were also found in circulation. Furthermore, sporadic cases of carbapenem-resistant *E. kobei* (*n* = 2), *Enterobacter sichuanensis* (*n* = 1), *Enterobacter roggenkampii* (*n* = 1), and *Pseudenterobacter timonenensis* (*n* = 1) were also recorded in this study.

### AMR in CR-ECC

Among the 70 CR-ECC strains, 78.57% (*n* = 55) were pan-resistant to 19 antibiotics (imipenem, meropenem, ertapenem, doripenem, ceftaroline, cefepime, ceftazidime, cefotaxime, aztreonam, piperacillin-tazobactam, ceftazidime-avibactam, gentamicin, ciprofloxacin, levofloxacin, trimethoprim-sulfamethoxazole, chloramphenicol, tetracycline, ampicillin, and amoxycillin-clavulanic acid). Among the newer drugs, high-level resistance [minimum inhibitory concentrations (MICs) >256 µg/mL] to ceftazidime-avibactam, a novel carbapenemase inhibitor was found in all NDM-containing isolates. Colistin resistance (MIC 128 μg/mL) was found in 35.7% of strains. The study isolates showed maximum susceptibility toward minocycline (92.9%), followed by tigecycline (85.7%), doxycycline (78.6%), and amikacin (75.7%). MIC_50_ and MIC_90_ for different antibiotics are shown in Table 1.

**TABLE 1:** MIC ranges, MIC_50_, and MIC_90_ of different antimicrobials in *E. cloacae* complex isolates (*n* = 70)

Antimicrobial agents	MIC (µg/mL)			
	Range of MIC	MIC_50_	MIC_90_	% of resistance
Carbapenems				
Imipenem	4–≥256	8	128	100
Meropenem	4–≥256	16	64	100
Ertapenem	8–≥256	32	128	100
Doripenem	4–512	16	64	100
Biapenem^*b*^	4–128	8	32	100
Cephems (cephalosporins)				
Ceftazidime	512–≥1,024	≥512	≥1,024	100
Cefotaxime	256–>1,024	512	1,024	100
Cefepime	16–512	64	512	100
Ceftaroline	8–64	16	32	100
Monobactams				
Aztreonam	32–≥512	256	512	100
β-Lactam combination agents				
Amoxicillin-clavulanic acid	≥128/64–512/32	128/32	256/64	100
Piperacillin-tazobactam	≥32/4–512/4	128/4	512/4	100
Ceftazidime-avibactam	≥16/4–128/4	ND^*a*^	ND^*a*^	52(74.28)
Aminoglycosides				
Gentamicin	32–128	32	64	100
Amikacin	8–>1,024	≥512	≥1024	17(30.90)
Arbekacin^*b*^	4–512	ND^*a*^	ND^*a*^	15(21.42)
Fluoroquinolones				
Ciprofloxacin	32–256	64	128	100
Levofloxacin	2–512	4	16	100
Folate pathway antagonists				
Trimethoprim-sulfamethoxazole	8/152–256/4,864	32/608	64/1,216	100
Phenicol				
Chloramphenicol	256–512	512	512	100
Tetracyclines				
Tetracycline	32–256	64	128	100
Doxycycline	0.5–64	32	64	15(21.42)
Minocycline	0.5–16	ND^*a*^	ND^*a*^	5(7.14)
Tigecycline	0.125–16	ND^*a*^	ND^*a*^	10(14.28)
Lipopeptides				
Colistin	≤0.25–512	ND^*a*^	ND^*a*^	25(35.71)
Polymyxin-B	≤0.125–512	ND^*a*^	ND^*a*^	26(37.14)

^*a*^ ND, not done. The MIC_50_ and MIC_90_ for arbekacin, ceftazidime-avibactam, colistin, minocycline, and polymyxin B were not done as a smaller number of isolates with high MIC may skew the final calculation.

^*b*^ Although MIC of arbekacin and biapenem was done in this study, their breakpoints are yet not available in CLSI-2022.

### Genotypic determinants in CR-ECC

#### Carbapenem resistance

**Fig 1:**
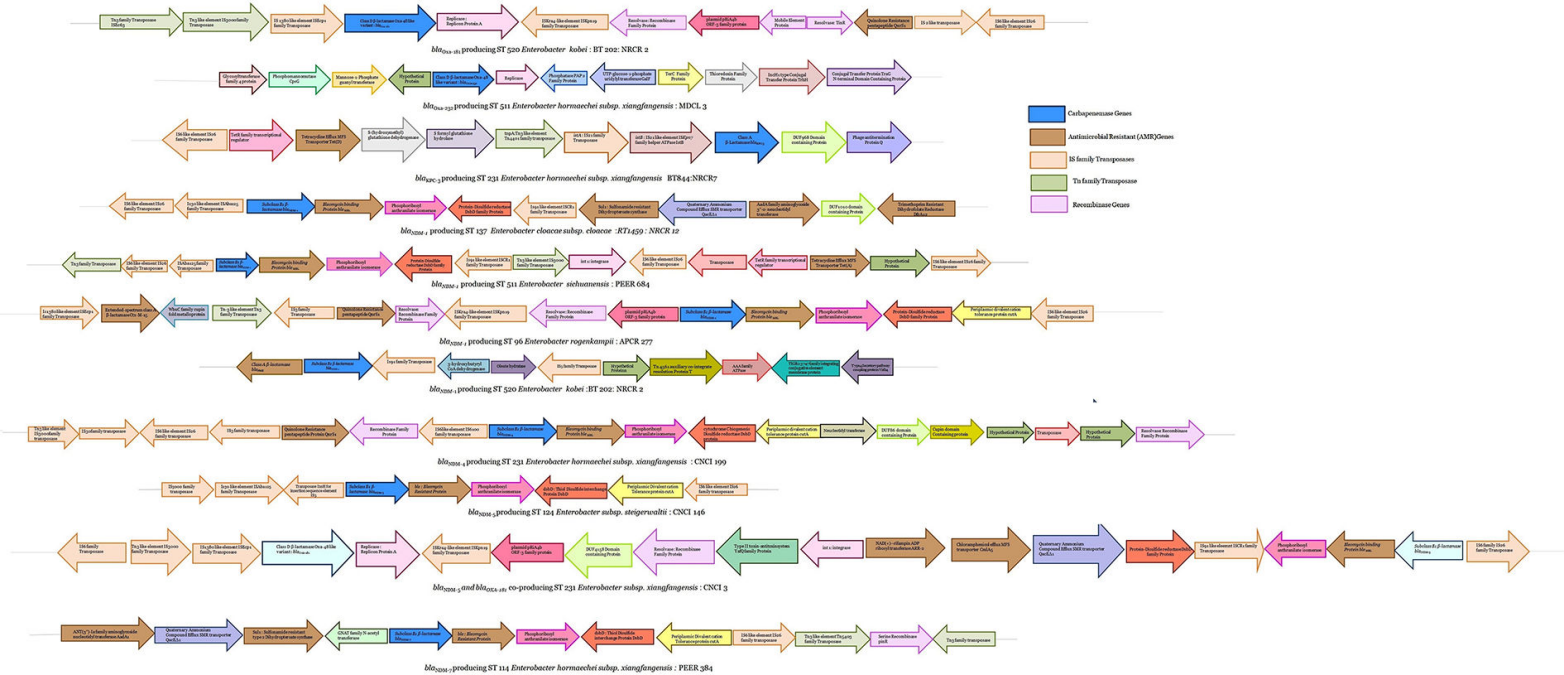
Schematic representation of the genetic environment of the various carbapenemases (*bla*_oxa-48_-like variant, *bla*_KPC_, and *bla*_NDM_) identified in our study.

Fifty-three of the 70 study strains were found to be carbapenemase producers with *bla*_NDM_ (*n* = 46) being the predominant, followed by *bla*_Oxa-48_-like variant (*n* = 9). Additionally, one isolate was found to be a *bla*_KPC_ (KPC-3) producer. The most common *bla*_NDM_ found in the study was *bla*_NDM-1_ (*n* = 36), followed by *bla*_NDM-5_ (*n* = 5), *bla*_NDM-7_ (*n* = 4), and *bla*_NDM-4_ (*n* = 1). The different bla_OXA-48_-like variants found in the study strains were *bla*_OXA-181_ (*n* = 7) and *bla*_OXA-232_ (*n* = 2). A total of six study strains were found to be coproducers of *bla*_NDM_ and *bla*_oxa-48_-like variants. No additional carbapenemase genes were found. At least two different carbapenemase genes were present in eight strains. The genetic environment of the different carbapenemase genes is depicted in Fig. 1.

#### ESBL and ampC genes

The *bla*_CTX-M-15_ (*n* = 19) was the principal ESBL gene found in the study isolates. Besides, *bla*_OXA-10_ (*n* = 3) and *bla*_OXA-21_ (*n* = 1) were also found. Notably, all the ESBL genes co-existed with carbapenemase genes. The *bla*_SFO-1_ gene was detected in five CR-ECC isolates, of which three were found in *E. hormaechei* subsp*. xiangfangensis*. All five *bla*_SFO-1_ positive isolates co-harbored *bla*_NDM-1_ and/or *bla*_OXA-232_ genes.

The *bla*_ACT_ [ACT-16 (*n* = 31), ACT-7 (*n* = 4), ACT-15 (*n* = 4), ACT-9 (*n* = 1), and ACT-14 (*n* = 1)] was the major AmpC gene found in study isolates. The *bla*_CMH-3_ gene (an uncommon AmpC type gene) was found in seven CR-ECC isolates with or without cohabitation of the *bla*_NDM-1_ gene.

#### Other resistance genes and integrons

Among the 16S methyltransferase genes conferring resistance to aminoglycosides, nine isolates were positive for *armA,* while three isolates were co-producer of *armA* and *rmtB*. The fosfomycin resistance (*fosA*) and plasmid-mediated quinolone resistance (PMQR) (*aac-(6′)-Ib-cr*) genes were uniformly present in all study isolates. In some isolates *qnrS or qnrB* were also present along with *aac-(6′)-Ib-cr* and/or mutation in *gyrA* and/or *parC* genes. The quinolone resistance-determining regions (QRDR) mutations detected were S83I (*n* = 6), S83F (*n* = 5), S83Y (*n* = 3), S83L (*n* = 1), and/or D87A (*n* = 7) in *gyrA* and S80I (*n* = 14) and S80R (*n* = 4) in *parC*.

**Fig 2:**
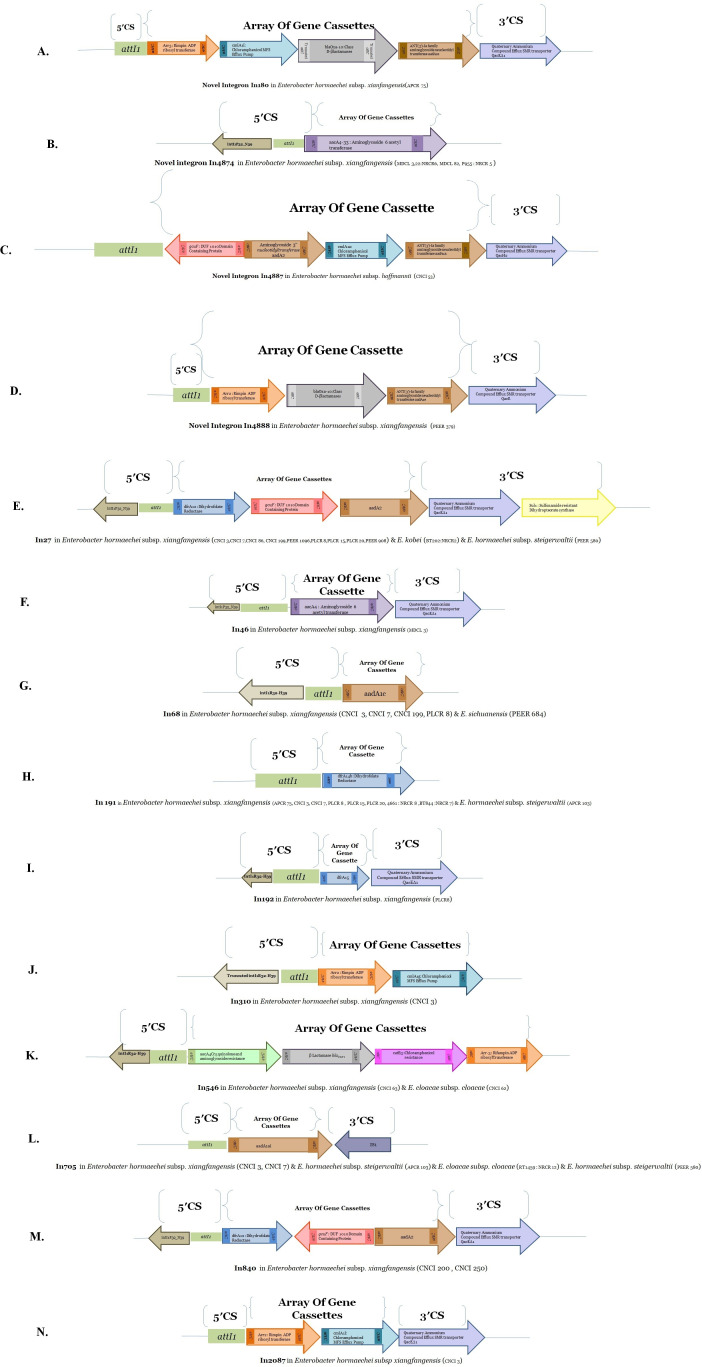
Schematic depiction of the class 1 integron with respective gene cassette arrays discovered in our study among ECC isolates resistant to carbapenems. INTEGRALL (http://integrall.bio.ua.pt/) curators validated and assigned integron numbers to each integron. In our CR-ECC isolates, we discovered four novel integrons (In180, In4874, In4887, and In4888).

Class 1 integrons were detected in all isolates. Eleven gene cassettes of various sizes were found, as shown in Fig. 2A through M. None of the carbapenemases or ESBLs (except *bla*_OXA-10_) genes were located within the integrons. Each of the class 1 integron was curated, validated, and assigned individual integron (In) number by the INTEGRALL curator (http://integrall.bio.ua.pt/). Notably, four novel integrons In180 (*arr2-cmlA1I-bla_O-10_-aadA1e*), In4874 (*aacA4*), In4887 (*dfrA12-gcuF-aadA2-cmlA1a-aadA1a-qacH2*), and In4888 (*arr2-bla_Oxa-10_-aadA1e*) were discovered in *E. xiangfangnensis* (*n* = 3) and *E. hoffmannii* (*n* = 1) strains (Fig. 2A through D).

### Plasmid analysis

**Fig 3:**
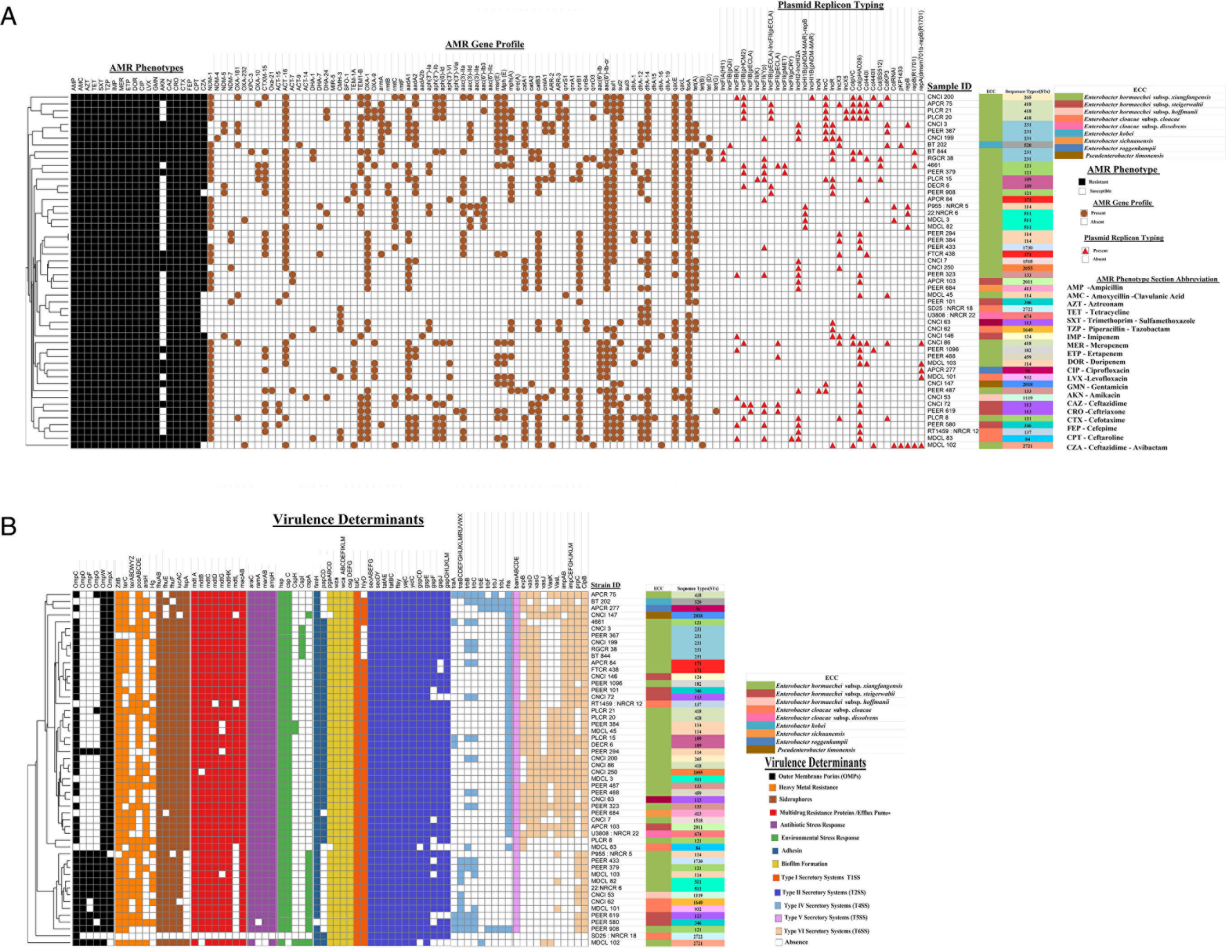
(A) Heatmap of CR-ECC study isolates depicting respective AMR phenotype, AMR gene, plasmid replicon typing, and sequence types. Cluster analysis was performed on the heatmap using Euclidean distance. (B) Heatmap depicting the presence or absence of different virulence determinants found in CR-ECC study isolates.

Ninety-six percent (*n* = 67/70) of the CR-ECC study isolates harbored ≥1 plasmid and showed great diversity in their profiles. Of these, plasmids of ≥70 kb in size were found to be transferable. Plasmid incompatibility typing identified nearly 19 plasmid types (Fig. 3) in circulation, of which IncFIB (*n* = 16), IncFII (*n* = 15), IncX3 (*n* = 10), IncHI1-HI2 (*n* = 9), IncC (*n* = 9), and IncR (*n* = 9) were the most prevalent. However, no association between plasmid types and particular AMR gene profiles was observed.

### Virulence determinants

The study isolates possessed the biofilm-forming *wcaABCDEFKLM*, *wza*, and *csgABCEFG* genes, while other related genes like *wzb*, *wzc*, or manC genes were absent. The characteristic aerobactin (siderophore) genes like *fhuAB* and *iucAB* were present in all study isolates but the complete operon (*fhuABCD* and *iucABCD*) was missing. The heavy metal resistance genes like *arsH* (arsenic), *pcoABCDEF* (copper), *terABCDWYZ* (tellurite), and *zitB* (zinc) were found in all strains. Environmental stress response genes (*clpB*, *hsp*, *cspCE*, and *ompR*) and antibiotic stress response genes (*marAB*, *araC*, and *ramA*) were detected in all CR-ECC strains. Notably, the carbapenemase-producing ECC strains were found to be lacking the efflux pump regulator *soxSR* gene. All except two isolates carried the genes *mdtABCDGHIJKL*, *macA*, and *macB* which mediate inherent resistance to a wide variety of drugs. In addition, genes like *fimH* and *papCD*, which help in adhesion to extracellular matrix were also present. All carbapenem-producing isolates carried the T6SS (*impABCDEFGHJKM*, *vasDJ,* and *evpB*) in addition to the T1SS (*hlyD* and *tolC*), T2SS (*ftsY*, *tatABC*, *yajC*, and *yidC*), T4SS (*traABCDEFGHIJKLMUW*, *trbBCJL,* and *rhs*), and T5SS (*bamABCD*). The presence or absence of the virulence determinant genes is depicted in Fig. 3B.

### Phenotypic detection of the role of efflux pump in AMR

The MIC of ertapenem showed a fourfold decrease or more in the presence of all three efflux pump inhibitors (NMP and PAβN) tested (Table 2), suggesting the existence of an association between efflux pump and carbapenem resistance.

**TABLE 2:** Effect of efflux pump inhibitor PAβN on different antibiotic susceptibilities and fold change in efflux pump, regulators, and OMP by RT-PCR

CR-ECC isolates	Cefepime MIC (µg/mL)		Ceftazidime MIC (µg/mL)		Ertapenem MIC (µg/mL)		Doripenem MIC (µg/mL)		RT-PCR fold change									
									RND pumps			Regulator of RND pumps			ampC	Outer membrane porins		
	−PAβN	+PAβN	−PAβN	+PAβN	−PAβN	+PAβN	−PAβN	+PAβN	acrA	acrB	tolC	ramA	soxS	soxR		mpC	mpD	mpF
**P2253: NRCR 13**	32	8	>1,024	32	4	1	4	1	0.651 ±0.927	7.511 ±1.997	2.376 ±0.561	1.234 ±0.957	53.980 ±1.365	6.922 ±1.657	6.712 ±1.245	20.125 ±1.247	8.102 ±0.893	1.37 ±1.281
**APCR 260**	32	4	>1,024	64	8	1	8	2	0.597 ±1.058	10.337 ±1.405	0.102 ±0.325	1.441 ±0.732	0.173 ±0.022	0.435 ±0.107	7.259 ±0.457	23.337 ±1.248	0.262 ±0.247	1.012 ±0.948
**U3306: NRCR 11**	32	8	>1,024	32	16	4	4	1	7.729 ±0.991	113.444 ±1.435	0.690 ±0.686	2.336 ±0.885	10.432 ±0.832	1.575 ±0.165	8.227 ±1.098	136.569 ±1.721	5.143 ±1.553	2.328 ±1.847
**U3639:** **NRCR 14**	64	8	>1,024	16	16	2	16	4	1.208 ±1.460	48.452 ±1.943	1.073 ±0.271	0.462 ±0.160	0.690 ±0.034	0.755 ±0.086	11.510 ±1.334	12.157 ±0.734	1.389 ±0.359	0.948 ±0.832
**4: NRCR 3**	16	4	>1,024	64	16	4	4	0.5	7.815 ±1.898	67.743 ±1.893	6.669 ±0.569	1.420 ±0.630	28.880 ±1.671	35.449 ± 1.386	12.755 ±1.119	0.835 ±0.030	0.253 ±0.095	0.195 ±0.120
**U3262: NRCR 23**	16	2	>1,024	64	8	2	4	0.25	5.374 ±1.382	7.796 ±0.254	3.749 ±1.234	5.387 ±1.282	5.981 ±0.127	0.458 ± 0.034	13.521 ±1.070	307.676 ±1.805	0.135 ±0.357	0.165 ±0.258
**CNCI 63**	16	4	>1,024	16	256	16	256	16	8.456 ±1.050	29.566 ±1.648	2.01 ±0.563	1.187 ±0.523	8.971 ±0.881	4.449 ± 0.353	11.201 ±1.881	116.759 ±1.885	25.860 ±1.990	11.170 ±0.0638
**SD25**	32	4	512	16	8	2	16	2	0.054 ±0.012	0.483 ±0.215	2.38 ±0.310	1.211 ±0.247	4.002 ±0.010	1.498 ± 0.092	8.992 ±0.989	6.942 ±0.456	0.503 ±0.010	44.663 ±1.883
***E.cloacae* ATCC 13047**	0.25	ND^*a*^	0.5	ND^*a*^	0.012	ND^*a*^	0.25	ND^*a*^	1	1	1	1	1	1	1	1	1	1

^*a*^ ND, not done; RT-PCR, reverse transcription-PCR.

### Western blotting

**Fig 4:**
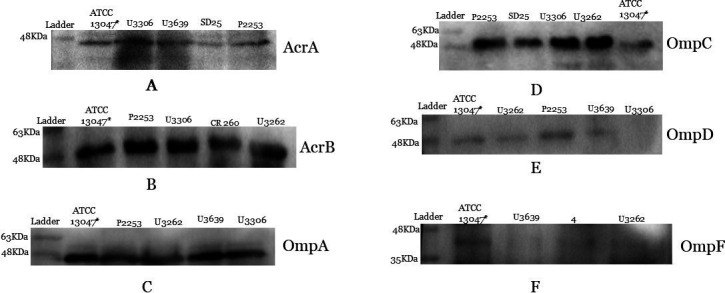
Western blot of AcrA, AcrB, and outer membrane porin (Omp) protein expression in CR-ECC isolates that do not produce carbapenemases. *Enterobacter. cloacae* ATCC 13047 was used as a control strain. (**A**) The AcrA protein in the U3306 isolate displayed increased protein expression in comparison to the control strain, while the SD25 isolate displayed decreased band intensity. The AcrA levels in isolates U3639 and P2253 were unchanged. (**B**) In the case of AcrB, all four test samples showed increased protein intensity when compared to the control. (**C**) No differences were observed between test and control strains for OmpA expression. (**D**) The expression of porin OmpF was completely lost in test strains. (**E**) The test strains showed overproduction of OmpC. (**F**) Complete loss of OmpD was found in U3306, along with decreased expression in U3262 and U3639, and increased expression in P2253 as compared to control.

The protein levels of AcrA and AcrB were found to be higher than the control in CR-ECC isolates. Among the OMPs, increased levels of OMP C and lower levels of OMP D were observed in some isolates. Furthermore, loss of OMP F and unchanged levels of OMP A were found in all isolates (Fig. 4; Fig. S1).

### Real-time PCR of detection of the role of efflux pump genes, porins, and ampC in AMR

Reverse transcription (RT-PCR) of the efflux pump gene (*acrAB, tolC*) and regulatory genes (*soxR, ramA*) showed significantly elevated levels of gene expression (Table 2), validating the phenotypic association between AMR and efflux pump in CR-ECC. Furthermore, all strains showed increased *ampC* gene expression. Additionally, *ompC* was more highly expressed in 29 ECC isolates than in the control ATCC13047, while *ompF* was significantly less expressed in these ECCs. No change in the expression of *ompA* was observed in the study isolates.

### Multi-locus sequence typing

**Fig 5:**
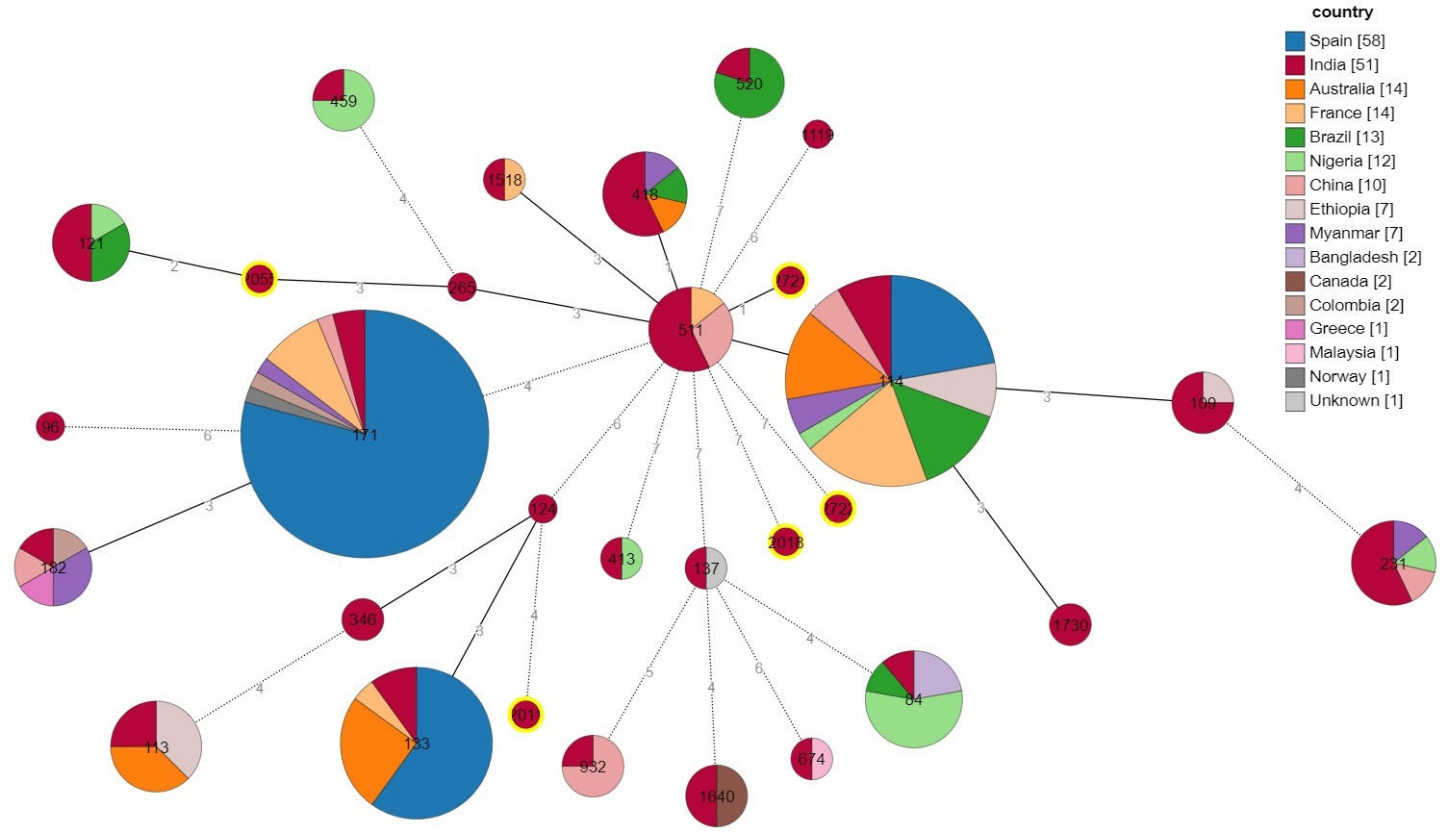
Minimum spanning tree based on MLST allelic profile: pictorial depiction of genetic relationship of different STs of ECC-CR clinical isolates found in this study by goeBURST and PHYLOViZ 2.0. Each kurtosis or node (circle) corresponds to one ST designated by a particular number, and the size of the circle is proportional to the number of isolates (genomes). Each circle is divided into cross-sections of different colors. Each color represents the different countries in which the STs are found (genomes downloaded from the pubMLST database, last accessed on 25 September 2023). The numbers on the branches represent the allelic differences. Branches with a length greater than three locus variants are outlined in dashes. All the STs obtained in this study (India) are represented in red color; all have been validated and curated by the pubMLST curator, and this is now available on the pubMLST website. The red-colored circles with yellow halo (ST2011, ST2018, ST2055, ST2721, and ST2722) depict the novel STs discovered in this study. Although ST96, ST124, ST346, and ST265 are represented only in red color, these are not exclusive to India but rather our study. This representation is because the pubMLST has only the Sanger sequence but no whole genome records for these STs.

The CR-ECC study isolates were assigned into 30 distinct STs by multi-locus sequence typing (MLST), namely ST114 (*n* = 6), ST231 (*n* = 5), ST418 (*n* = 4), ST511(*n* = 3), ST171, ST121, and ST133 (*n* = 2 each) (Fig. 5). Of these, ST2011 identified in *E. hormaechei* (*bla*_NDM-1_ producer) and ST2055 identified in *E. hormaechei* (*bla*_NDM-7_ producer); ST2018 in *Pseudenterobacter timonensis* (*bla*_NDM-1_ producer), ST2721 in *E. hormaechei* (*bla*_OXA-232_ producer), and ST2722 in *E. cloacae* (no carbapenemsase producer) were novel. Analysis of the molecular epidemiological relationships between different STs by goeBURST revealed that ST114, ST418, and ST511 were closely related as there was only a single-locus variation among them with ST114 being the central ST. The novel ST2721 originated from ST511, while ST2055 emerged from ST121 through single and double locus variation, respectively. All other STs were singletons (Fig. 5).

### Whole genome-based MLST

**Fig 6:**
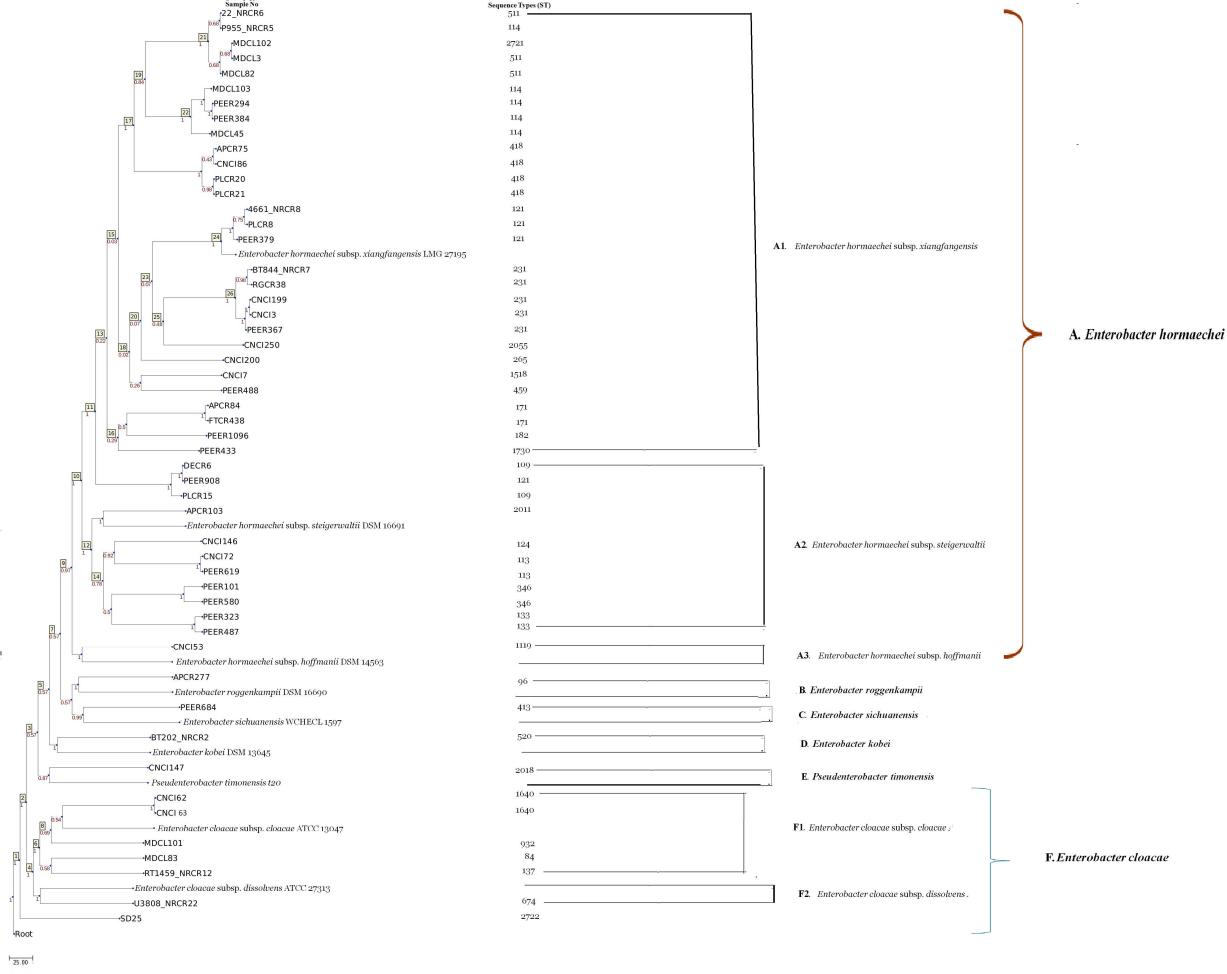
wg-MLST-based phylogenetic tree of CR-ECC strains. The reference genomes of respective ECC type (*E. hormaechei* subsp. *xiangfangensis* LMG 27195 accession no. CP017183; *E. hormaechei* subsp. *steigerwaltii* DSM 16691 accession no. CP017179; *E. hormaechei* subsp. *hoffmanii* DSM 14563 WJWQ00000000; *E. roggenkampii* DSM 16690 accession no. CPO17184; *E. sichuanensis* WCHECL1597 accession no. POVL00000000, *P. timonensis* mt20 accession no. FCOP00000000; *E. kobei* DSM 13645 accession no. CP017181, *E. cloacae* ATCC 13047 accession no. CP001918, and *E cloacae* subsp. *dissolvens* ATCC 23373 accession no. WJWQ00000000) were used to perform the wg-MLST.

The whole genome-based MLST (wg-MLST) cladogram segregated *E. hormaechei*, *E. roggenkampii, E. sichuanensis, E. kobei, P. timonensis,* and *E. cloacae* into separate clusters A, B, C, D, E, and F, respectively (Fig. 6). Three distinct sub-clusters A1, A2, and A3 were observed within Cluster A representing *E. hormaechei* subsp. *xiangfangensis*, subsp. *steigerwaltii,* and subsp. *hoffmanii,* respectively, while cluster F was sub-divided into sub-cluster F1 (*E. cloacae* subsp. *cloacae*) and F2 (*E. cloacae* subsp. *dissolvens*). The cladogram of *P. timonensis* was related to other ECC strains in this study, which was surprising as this species is no longer a part of the ECC family (3).

### Pulsed-field gel electrophoresis

**Fig 7:**
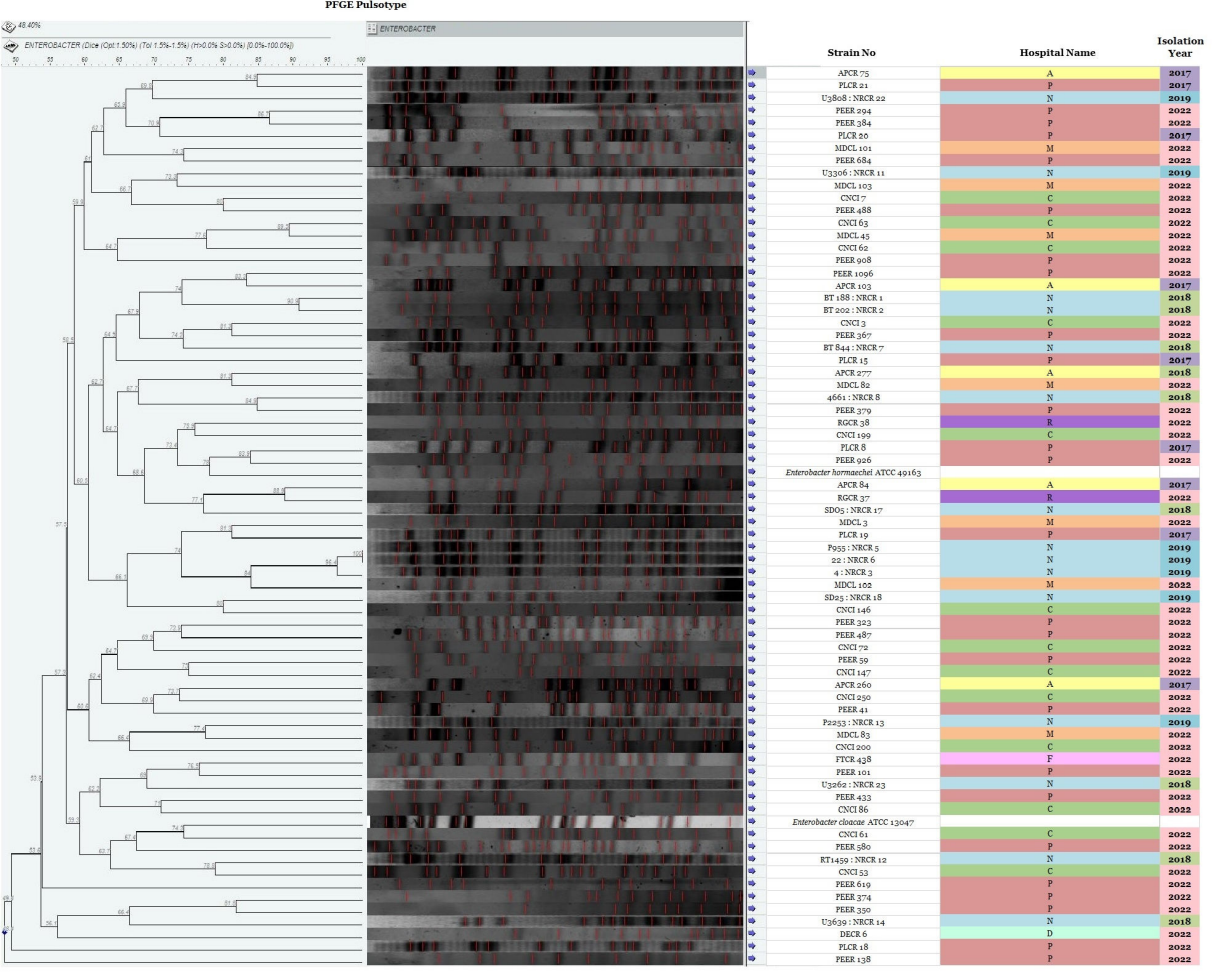
Dendrogram of PFGE cluster analysis of *Xba*I-digested patterns of 70 carbapenem non-susceptible ECC isolates along with their year of isolation and hospital names (abbreviation).

Restriction digestion of 70 isolates with *Xba*-I revealed the presence of 69 pulsotypes in pulsed-field gel electrophoresis (PFGE) with a similarity coefficient of 48.40% (Fig. 7). No correlation was found among the strain pulsotype and hospital. There was no predominance of any pulsotype in any hospital. However, only one pair of isolates (P955: NRCR 5 and 22: NRCR 6) was found to be clonal (100% similarity), which was isolated in 2019 from the same hospital (Hospital N) but in different months and from different patients. The phylogenetic diverse ECC strains in circulation suggest diverse reservoirs of infection and enhanced risk of dissemination of CR-ECC strains.

## DISCUSSION

In India, no comprehensive study solely focused on carbapenem-resistant ECC strains has been done in 15 years. A handful of studies on the *Enterobacteriaceae* family from southern India have mentioned the isolation of *E. cloacae* harboring *bla*_NDM-1_ (sample type was not specified) (16, 17) and *E. cloacae* possessing *bla*_NDM-1_ and *bla*_OXA-48_-like variants (*bla*_OXA-181_) from blood samples (17). One carbapenem-resistant *E. hormaechei* isolate has also been reported from western India (18). However, unlike this study, the previous studies from India have neither explored the detailed mechanisms of carbapenem resistance in ECC nor engaged in any whole genome-based study. Additionally, the speciation of ECC strains causing BSI and their molecular types including multi-locus sequence types were not mentioned in any of the previous studies carried out in India.

In this study, *E. hormaechei* was found to be the major blood isolate among all the *Enterobacter* sp. surpassing *E. cloacae* which was in agreement with earlier studies globally (19). To the best of our knowledge. This is the first study from India reporting the emergence of carbapenem, colistin, and ceftazidime-avibactam resistance in *E. xiangfangensis* among the ECC complex.

A diverse array of carbapenemases were found in our study (Fig. 1). Both intraspecies and interspecies difference was observed in the genetic environment (upstream and downstream) of all the carbapenem genes identified in this study. This was mainly due to differences in the insertion sequence (IS) elements, transposons, and recombinase genes. The Indian subcontinent is a major hub (20) for *bla*_NDM_ carbapenemase, and consequently, the *bla*_NDM-1_ gene was found to be the dominant carbapenemase in this study. Earlier studies from China, Brazil, and Lebanon have also reported *bla*_NDM-1_ in *E. hormaechei* species (19). It is noteworthy that besides *E. hormaechei and E. cloacae, bla*_NDM-1_ was also discovered in *E. kobei*, *E. sichuanensis, E. roggenkampii,* and *P. timonensis* in this study, which is the first of its kind from India (Fig. 3). In addition, *bla*_NDM-4_ and the rare allele *bla*_NDM-7_ were also identified in *E. hormaechei*, which was previously reported from Spain and Canada only (19). The presence of *bla*_NDM-5_ in *E. xiangfangensis and E. steigerwaltii* strains was also not previously reported in India.

**Fig 8:**
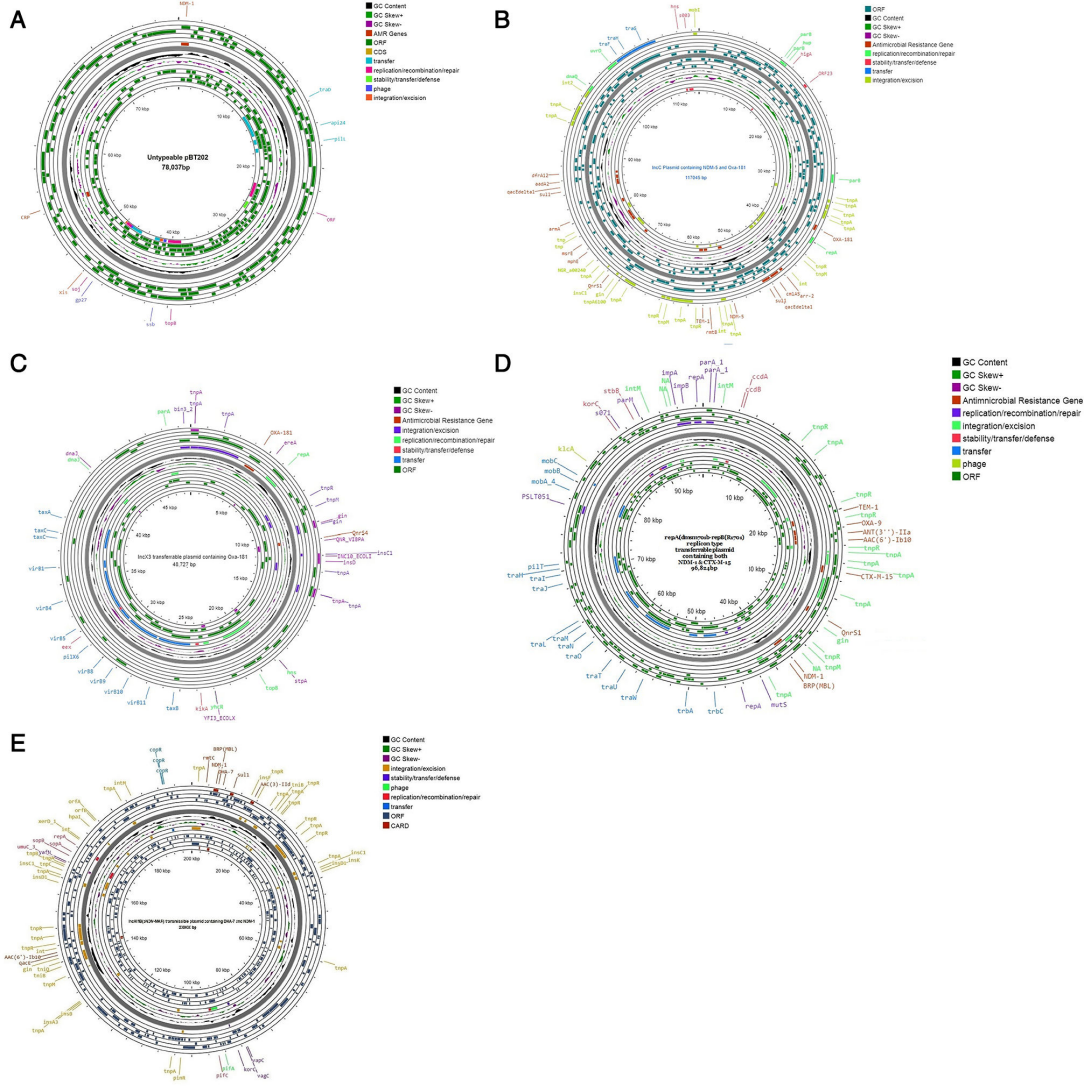
Circular representations of transmissible plasmids harboring different carbapenemase genes. (**A**) Untypeable transmissible plasmid harboring *bla*_NDM-1_ in *E. kobei* (ST 520). (**B**) IncC replicon-type transmissible plasmid co-harboring *bla*_NDM-5_ and *bla*_OXA-181_ in *E. hormaechei* subsp. *xianfangensis* (ST 231). (**C**) IncX3 replicon-type transmissible plasmid harboring *bla*_OXA-181_ in *E. hormaechei* subsp. *xianfangensis* (ST 121). (**D**) repA(dmsm701b)-repB(R1701) replicon-type transmissible plasmids co-harboring *bla*_NDM-1_ and *bla*_CTX-M-15_ in *E. roggenkampii* (ST 96). (**E**) IncH1B(pNDM-MAR) replicon-type transmissible plasmid co-harboring *bla*_NDM-1_ and *bla*_DHA-7_ *in E. hormaechei* subsp. *xianfangensis* (ST 511).

The ECC strains with *bla*_KPC_ gene alleles have been geographically restricted to north, south, and central America (19). In this study, we identified *bla*_KPC-3_-producing *E. xiangfangensis,* which has not been previously reported from India, although it was reported in *E. hormaechei* in the USA (19). Interestingly, this isolate also harbored *bla*_NDM-1_ and both these genes were transmissible but via two different plasmids of IncC and IncF2 types, respectively. A novel 78 kb untypeable pBT202 plasmid (Fig. 8A) carrying *bla*_NDM-1_ in *E. kobei* was a surprise finding.

The *bla*_OXA-48_-like variants carbapenemases are not very frequently reported in *Enterobacter sp*., although few variants have been reported in *E. steigerwaltii* and *Enterobacter oharae* from middle-eastern countries, Vietnam, Belgium, and Brazil in the past (19). In this study, we report emergence of *bla*_OXA-181_ and *bla*_OXA-232_ in *E. xiangfangensis* and *E. kobei*, which has not been previously documented in *Enterobacter* sp. This is also the first report from India demonstrating the co-transmission of *bla*_NDM-5_ and *bla*_OXA181_ via the same plasmid (IncC) in *E. xiangfangensis* (Fig. 8B). The conjugative IncX3 plasmid of 48 kb size carrying *bla*_OXA-181_ gene in *E. kobei* was also unique (Fig. 8C), confirmed by Plasmid Finder curator.

Among the ESBL genes, *bla*_CTX-M_ (CTX-M-2, 3, 9, 14, and 15) and *bla*_SHV_ (SHV-12, 78, and 236) were very commonly reported in Enterobacter globally (19). In this study, 19 strains harbored the *bla*_CTX-M-15_ gene, which were mostly carried by IncF2 and IncHI1 plasmid types (Fig. 3). However, we discovered a novel 96.8 kb transmissible plasmid *repA* (dmsm701b-repB(R1701) in *E. roggenkampii* (Fig. 8D) which co-carried *bla*_CTX-M-15_ and *bla*_NDM-1_ genes. Amid the rare ESBL types reported in *Enterobacter* spp., the *bla*_SFO-1_ gene was found to be endemic in Asian (China, Vietnam, and Japan) countries (19). In tandem with this *bla*_SFO-1_ gene was identified in *E. xiangfangensis and E. cloacae* strains. However, it was successfully transferred alongside *bla*_NDM-1_ in only *E. xiangfangensis* strains by two different plasmids (IncFII and an untypeable plasmid).

The study strains also harbored rare AmpC genes like *bla*_CMH-3_ and *bla*_ACT_. The chromosomal *bla*_CMH-3_ gene with/without carbapenemases was found only in *E. cloacae* strains, supporting a Spanish study (19). Alleles of the *bla*_ACT_ gene (ACT-16, ACT-7, ACT-15, and ACT-9) were found in both carbapenemase-producing and non-carbapenemase-producing (non-CP-ECC) study isolates. Most of the *bla*_ACT_ gene variants in *Enterobacter* spp. were recorded from Asia–Pacific regions (19, 21). Only *E. kobei* had the species-specific intrinsic ampC *bla*_ACT-9_, as previously reported (19). *bla*_ACT-16_ was found in different ECC subsp., but *bla*_ACT-15_ and *bla*_ACT-7_ were only found in *E. steigerwaltii* strains (Fig. 3). We found three *bla*_DHA_ alleles (DHA-1, DHA-7, and DHA-24) co-existing with *bla*_NDM-1_, unlike earlier Chinese studies that documented only *bla*_DHA-1_ (19). A 200 kb IncHI1B (pNDM MAR) conjugative plasmid co-containing *bla*_NDM-1_ and *bla*_DHA-7_ was found in *E. xiangfangensis* (Fig. 8E). We also found *bla*_MIR-5_ and *bla*_NDM-1_ in two *E. xiangfangensis* isolates for the first time.

In non-CP-ECC strains (*E. cloacae* subsp. *cloacae*), resistance to carbapenem was due to increased expression of efflux pump genes (*acrA-acrB* and its regulator *soxS/ramA*), along with elevated expression of *ampC* cephalosporinase, upregulation of *ompC,* and downregulation of *ompF* (Fig. 4). These findings substantiated earlier reports, which suggested that a decreased expression of *ompF* could result in a compensatory increase in *ompC* expression (21, 22).

This study identified a plethora of STs (*n* = 30) not previously reported from India (Fig. 5), which was supported by STs from China and the USA (9, 21, 23). The findings of our study demonstrate an extensive variety of CR-ECC strains and variations in genetic relatedness, in line with previous research (24). The most epidemic STs in ECC worldwide were ST114, ST171, and ST78, but the predominating ST varied by country and region (9, 21). None of the STs were predominated in this study. The only subsp.-specific ST in this study was ST520, which was previously reported for *E. kobei* (8). STs are usually linked to an AMR phenotype, but this study found no such association. Four common STs associated with ESBLs in *Enterobacter* spp. worldwide are ST66, ST78, ST108, and ST114, all of which carry *bla*_CTX-M-15_ (9). Out of these four common ESBL STs, only ST114 was found in three *E. xiangfangensis* study isolates. Two of these isolates had *bla*_NDM-7_ and *bla*_CTX-M-15_ on a transmissible IncX3 plasmid, while another ST114 isolate did not. ST171 has become the most common CR-ECC clone in the USA due to the widespread use of carbapenems and fluoroquinolones. Most ST171 isolates carry *bla*_KPC-3_, but some may carry *bla*_KPC-2_ or *bla*_KPC-4_(9, 24). Interestingly, two ST171 isolates in this study carried the *bla*_NDM-1_ gene on IncX3 or IncFII (Yp) plasmids instead of *bla*_KPC_ genes. PFGE was found to be more discriminatory than MLST. Similar to earlier reports, the study isolates with identical STs had different pulsotypes, suggesting the circulation of diverse clones of *Enterobacter* spp. in this region. Unlike PFGE dendrogram, wg-MLST clustered ECC study strains from the same subspecies into the same clades more efficiently (Fig. 6).

There are a number of limitations in this study. The sample collection was limited to a few hospitals in Kolkata, so it may not accurately represent CR-ECC across India. The hybrid assembly could not be performed for all isolates, limiting plasmid mapping analysis for all CR-ECC isolates. WGS identified a number of virulence factors in *Enterobacter* spp*.*, but expression levels of virulence genes were not evaluated as it was beyond the scope of this study.

### Conclusion

The CR Enterobacterales family causes most nosocomial infections worldwide. WGS analysis identified *E. xiangfangensis* as the major CR-ECC pathogen. However, clinical microbiology laboratories cannot accurately identify ECC at the species level. Variations in study methods may also skew results. Sequencing-based identification studies are crucial because pan-resistant nosocomial pathogens may usher in a “post-antibiotic era.” In ECC study isolates, carbapenemase (*bla*_NDM_), overexpression of ESBL (*bla*_CTX-M-15_), and/or AmpC (*bla*_DHA_ and *bla*_ACT_), and porin loss were the main mechanisms of carbapenem resistance. WGS could detect emerging and/or low-incidence genes like *bla*_SFO-1_ and *bla*_CMH-3_ that were not routinely monitored for AMR. CR-ECC’s diversity and high plasmid uptake suggest a specialized antibiotic resistance prevention strategy.

## MATERIALS AND METHODS

### Strain collection and identification

CR-ECC strains were isolated from the blood of patients admitted to the intensive care unit of eight tertiary care hospitals in Kolkata from January 2017 to December 2022. Patients had not received any carbapenem antibiotic prior to hospital admission. Pure cultures of the isolates were sent to the bacteriology division at ICMR-NICED after they had been identified at the respective hospitals using the VITEK MS and VITEK 2 Compact system (bioMérieux, France). The speciation of ECC was confirmed at ICMR-NICED by both 16S rRNA PCR (6) and species-specific multiplex PCR (25).

### Antibiotic susceptibility testing and determination MIC

Antibiotic susceptibility testing (AST) was performed by Kirby–Bauer disk diffusion method (26) using 19 different antibiotic discs (BD Diagnostics, USA), namely ampicillin (10 µg), amoxicillin-clavulanate (20/10 µg), ampicillin-sulbactam (100/10 µg), piperacillin-tazobactam (100/10 µg), chloramphenicol (30 µg), tetracycline (30 µg), trimethoprim-sulfamethoxazole (1.25/23.75 µg), cefepime (30 µg), cefotaxime (30 µg), ceftazidime (30 µg), aztreonam (30 µg), imipenem (10 µg), meropenem (10 µg), doripenem (10 µg), ertapenem (10 µg), ciprofloxacin (5 µg), levofloxacin (5 µg), amikacin (30 µg), and gentamicin (10 µg). The broth microdilution method (BMD) was performed (26) to determine the MIC of the aforesaid antibiotics. Furthermore, MIC was also performed for an additional eight antibiotics (arbekacin, ceftaroline, colistin, polymyxin B, minocycline, doxycycline, biapenem, and ceftazidime-avibactam). The results of AST and MIC were interpreted following the Clinical and Laboratory Standards Institute (CLSI) guideline (CLSI, 2022) (27) and EUCAST 2022 (28) using *E. coli* ATCC 25922 strain and *Pseudomonas aeruginosa* ATCC 27853 as control. MIC_50_ and MIC_90_ were estimated as per standard protocol (29).

### Phenotypic detection of carbapenemases

Phenotypic detection of carbapenemases was carried out by Rapidec Carba NP-kit (bioMérieux, Marcy-l'Étoile, France) following the user’s manual. The production of carbapenemase by bacteria is indicated by a color change from red to yellow within 30 min.

### Genotypic characterization of AMR genes in terms of antibiotic resistance determinants

Molecular detection of different carbapenemases (Class A-KPC, IMI, SME, SPM, GES; Class B-VIM, IMP, NDM, SPM, GIM, SIM; and Class D-OXA-48 like variants) (30); ESBLs (TEM, SHV, and OXA-1/9); plasmid-mediated AmpC β-lactamases (MOX, CMY, DHA, ACC, MIR/ACT, and FOX) (31); 16S rRNA methylase-encoding genes (*rmtA, rmtB, rmtC, rmtD,* and *armA*) (32); PMQR genes (*qnrA*, *qnrB, qnrC, qnrD, qnrS, qepA,* and *aac(6′)-Ib-cr*) (33); and integrons (class 1, 2, and 3) were determined by multiplex PCR (34). Furthermore, singlex PCR and Sanger sequencing helped in determining the alleles of *bla*_NDM_, *bla*_KPC_, *bla*_Oxa-48_-like variants, *bla*_CTX-M_, *bla*_SHV_, *bla*_TEM_, *bla*_Oxa-ESBL_, *bla*_SFO_, and *bla*_CMH_ genes (Table S1). QRDR mutations in the *gyrA*, *gyrB*, *parC,* and *parE* responsible for quinolone/fluoroquinolone resistance were also elucidated by comparing with standard reference genomes obtained from https://www.ncbi.nlm.nih.gov/genome/.

### Determination of transferability of AMR genes by conjugation and characterization of transconjugants

The conjugal transfer of AMR genes from test strains (donor) to plasmid-free sodium azide-resistant *E. coli* J53 (recipient) was tested by liquid mating assay at 37°C. An isolated fresh colony of donor and recipient cells from Chrome Agar plates (Difco) was inoculated into separate tubes with 3 mL LB broth and incubated at 37°C for 18 h with shaking. Donor and recipient were subsequently mixed in fresh LB broth in a 1:5 ratio and incubated without shaking. After 18 h, cells were serially diluted and spread on chrome agar plates with 100 mg/L sodium azide and meropenem (2 mg/L) (Sigma-Aldrich, USA). After overnight culture, biochemical profiling confirmed transconjugants (TC). The MICs of antimicrobials were determined by BMD, and AMR determinants in the TC were confirmed by PCRs using TC plasmid DNA as a template.

### Determination of plasmid profiles and their replicon-typing

Plasmid DNA was isolated from the wild-type strains and the TCs by both the Kado–Liu method (35) and Qiagen Plasmid Midikit (Qiagen, Germany). The extracted plasmid DNA was electrophoresed in 0.8% agarose gel at 70 V using 1× TBE running buffer and stained with ethidium bromide (0.5 g/mL). Plasmid sizing was done by FP-Quest (Bio-Rad, USA) software using *E. coli* V517 and *Shigella flexneri* YSH6000 as molecular weight markers. Replicon-typing PCR elucidated the plasmid incompatibility types (36).

### Determination of the role of efflux pump in AMR

The role of the efflux pump in carbapenem resistance was determined phenotypically by measuring the MICs of ertapenem both in the absence and in the presence of efflux pump inhibitors (EPI) 1-(1-naphthylmethyl)-piperazine (NMP; 25 mg/L) and PAβN (1 mg/L) and (Sigma-Aldrich). MIC was determined by the BMD method and carried out in triplicates in separate experiments. A fourfold reduction or more in MIC in the presence of EPI was considered significant.

### Determination of carbapenem resistance in non-carbapenemase-producing CR-ECC isolates by western blot and RT-PCR

#### SDS-PAGE and immunodetection of efflux pump proteins (AcrA and AcrB) and outer membrane porins (OmpD, OmpC, OmpF, and OmpA)

Whole-cell extracts of isolates were resolved on 11% SDS-polyacrylamide gels with 20 µg of protein loaded per lane and then transferred to Immobilon-P filters (Millipore) in accordance with established protocols (37). Polyclonal anti-AcrA antibody (1:50,000 dilution), polyclonal anti-AcrB antibody (1:70,000 dilution), polyclonal anti-OmpC, polyclonal anti-ompF antibody (1:30,000 dilution), and polyclonal anti-OmpA antibody (1:30,000 dilution), polyclonal anti-ompD (1:50,000 dilution) (Biobharti Life Sciences Private Ltd., Kolkata) were applied to detect AcrA-AcrB and OMPs, respectively.

To eliminate any chances of an incorrect reading of protein levels in western blot, real-time PCR was subsequently performed to determine the expression levels of efflux pump and outer-membrane porins.

#### RT-PCR for determining efflux pump (acrA, acrB, and tolC) and their regulators (ramA, marA, and soxS) and outer membrane porin (OmpC, OmpD, and OmpF) gene expression

The bacterial isolates were cultured overnight at 37℃ in LB broth under antibiotic (meropenem) stress. Total RNA was extracted from the log-phase of two replicate cultures, using Trizol Reagent. Quantification and quality check of the extracted RNA was done using NanoDrop Biophotometer Plus (Eppendorf, Germany). Total RNA was digested with RNase-free DNaseI (New England Biolabs, USA) to remove any contaminating genomic DNA. Thereafter, cDNA was synthesized employing QuantiNova cDNA Synthesis Kit (Qiagen) and DNaseI-treated RNA as a template. To monitor gene expression levels, real-time RT-PCR using specific gene primers (as listed in Table S1) was carried out in Step One Plus Real-Time PCR System (Applied Biosystems). The 2^-ΔΔCT^ method (38) was used to calculate relative RNA expression levels, after normalizing with ribosomal housekeeping gene *rpoB*. All real-time RT-PCR experiments were repeated thrice, with *rpoB* gene as an internal control (39). Each target gene’s relative expression was then calibrated against the expression of an *E. cloacae* ATCC 13047 pan susceptible isolate (expression = 1). A twofold increase in gene expression in comparison to the reference strain was considered significant overexpression.

### Molecular typing method

#### MLST

The MLST of ECC based on seven “housekeeping genes” (*dnaA, fusA, gyrB, leuS, pyrG, rplB*, and *rpoB*) was performed as per protocol available on the MLST website (https://pubmlst.org/organisms/enterobacter-cloacae). The STs were assigned on submitting gene sequences to the MLST website (https://pubmlst.org/bigsdb?db=pubmlst_ecloacae_seqdef&l=1&page=sequenceQuery). The phylogenetic relationship between different STs was analyzed by goeBURST and PHYLOViZ 2.0.

#### PFGE

Clonality of the CR-ECC isolates was determined by PFGE following standard protocol (CDC, 2013), using *Xba*I (50 U/plug) restriction digestion enzyme and CHEF-DRIII pulsed-field electrophoresis systems (Bio-Rad), with switch times of 5 and 35 s, and run duration of 21 h. The *Xba*I*-*digested *Salmonella* Braenderup H9812 was used as a molecular weight ladder, while *Xba*I-digested *E. cloacae* ATCC 13047 and *E. hormaechei* ATCC 700323 were used as positive controls. FP-Quest v4.5 (Bio-Rad) generated the dendrogram based on the Dice coefficient with 1.5% position tolerance. The PFGE band patterns were interpreted as previously described (40).

### Whole genome shotgun sequencing analysis

The Qiagen DNeasy blood & tissue kit (Qiagen) was used for genomic DNA isolation. The DNA was quantified using Qubit 3.0. (Thermo Fisher Scientific, USA). Nextera XT was employed for DNA library preparation for paired-end sequencing (2 × 501 cycles) and sequenced on Illumina Novaseq 6000 platform using the protocol for v1.5 chemistry and a 2 × 250 bp read length (Illumina Inc., San Diego, CA).

Quality assessment of “paired-end raw reads” and “adapter trimming” was done by FastQC v0.11.9 (https://www.bioinformatics.babraham.ac.uk/projects/fastqc/) and Trimmomatic v0.39 (http://www.usadellab.org/cms/?page=trimomatic), respectively. The online software SPAdes v3.15.3 as well as CLC workbench premium 21.0 (Qiagen) were used for *de novo* assembly of raw reads. QUAST v5.0.2 (http://quast.sourceforge.net/) evaluated the quality of the assembled contigs. All of the incorrect mappings in the contigs generated during the assembly process were fixed by PILON v1.23. The RAST server (http://rast.nmpdr.org) helped in annotating the genomes and the .gbk file generated was viewed in Artemis (Sanger UK genome viewer). Genomic pictures were generated with the help of the SNAP Gene program (4.3.8). DFAST (https://dfast.ddbj.nig.ac.jp/) determined the genome characteristics. Species identification of *Enterobacter* spp. was done by pubMLST (https://pubmlst.org/species-id) and ANI calculator (41). Resfinder 4.1 (https://cge.cbs.dtu.dk/services/ResFinder/) identified the AMR genes. The INTEGRALL database (http://integrall.bio.ua.pt) helped in locating integrons within the genomes. PlasmidFinder 2.1 (http://cge.cbs.dtu.dk/services/PlasmidFinder/) determined the plasmid incompatibility (Inc) types present in Enterobacter genomes. Virulence determinants were analyzed using pathofact (https://pathofact.lcsb.uni.lu/). The wg-MLST-based phylogenetic dendrogram based on 209 loci was constructed using (https://github.com/cenesis/cano-wgMLST).

## Data Availability

All genome sequences have been submitted to the NCBI database under BioProject numbers PRJNA721536 and PRJNA970514.

## SUPPLEMENTAL MATERIAL

Fig. S1 spectrum.03529-23-s0001.tif Densitometry analysis of blots.

Table S1 spectrum.03529-23-s0002.doc Primers used in this study.

ASM does not own the copyrights to Supplemental Material that may be linked to, or accessed through, an article. The authors have granted ASM a non-exclusive, world-wide license to publish the Supplemental Material files. Please contact the corresponding author directly for reuse.
