# Supplementary figures and images for: Whole genome sequence-based molecular characterization of blood isolates of carbapenem-resistant Enterobacter cloacae complex from ICU patients in Kolkata, India, during 2017–2022: emergence of phylogenetically heterogeneous Enterobacter hormaechei subsp. xiangfangensis
Source: Microbiol Spectr. 2024 Feb 22;12(4):e03529-23. doi: 10.1128/spectrum.03529-23 (PMC10986559; doi:10.1128/spectrum.03529-23)

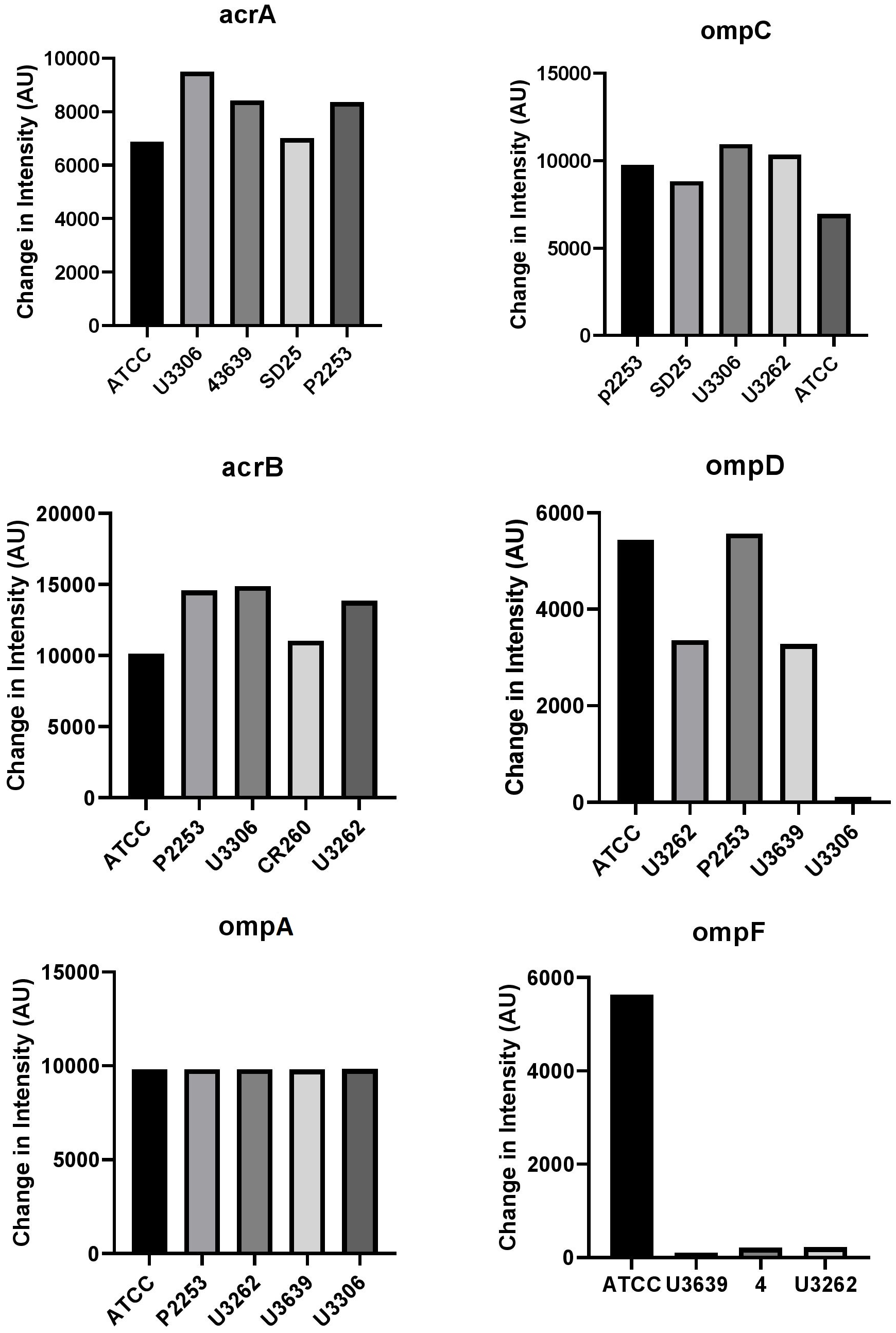

Supplement: Fig. S1 — Densitometry analysis of blots. [file spectrum.03529-23-s0001.tif]
