# Supplementary material for: Whole genome sequence-based molecular characterization of blood isolates of carbapenem-resistant Enterobacter cloacae complex from ICU patients in Kolkata, India, during 2017–2022: emergence of phylogenetically heterogeneous Enterobacter hormaechei subsp. xiangfangensis
Source: Microbiol Spectr. 2024 Feb 22;12(4):e03529-23. doi: 10.1128/spectrum.03529-23 (PMC10986559; doi:10.1128/spectrum.03529-23)
Supplement: Table S1 — Primers used in this study. [file spectrum.03529-23-s0002.doc]

| Carbapenemase | | | |
| --- | --- | --- | --- |
| *bla*_NDM_ | F--GAAGCTGAGCACCGCATTAG  R--GGGCCGTATGAGTGATTGC | 758bp | **This Study** |
| *bla*_KPC_ | F--ATCGCCGTCTAGTTCTGCTG  R--GCCCGTTGACGCCCAATC | 867bp |
| *bla*_oxa-48_like variant | F-- TATCGGCTGTGTTTTTGGTG  R-- CTTCTTTTGTGATGGCTTGG | 754bp |
| Other β-lactamase | | |
| *bla*_CTX-M_ | F--TGATTTTATTGAAAATGACCTCGT  R--AACCTAAGGCAGAAAGCCGT | 808bp |
| *bla*_CMH_ | F--GCACCGATGTCAGAAAAACA  R--ACCGGGTTCGGATAACTTTT | 1034bp |
| *bla*_TEM_ | F--CATTTTCGTGTCGCCCTTAT R--CATCCATAGTTGCCTGACTCC | 796bp |
| *bla*_Oxa-ESBL_ | F-- CAATCCGAATCTTCGCAATAC  R-- TCGATAGAGCGAAGGATTGC | 776bp |
| *bla*_SHV_ | F--TGCGTTATATTCGCCTGTGT  R-- GGCGATTTGCTGATTTCG | 821bp |
| *bla*_SFO_ | F-- TACGTCAAACCACCCTGATG  R-- ATTCTGCTGTGGCTGAGTGA | 812 bp |
| ampC β-lactamase | | |
| *bla*_ACT_ | F-- GCCTCTCTTGCTCTGCTCTC  R-- TTTATTCGCGAGCATCACAA | 1049bp |
| *bla*_DHA_ | F--AACACTGATTTCCGCTCTGC  R-- GCCTGTGCAGCTTTGACTCT | 1098bp |
| *bla*_MIR_ | F--GACAAAATCCCTAAGCTGTGC  R-- TTTATTCGCCAGCATCACAA | 1081bp |

**Table S1 : List of Primers used in this study**
